# An early clinical comparative study on total knee arthroplasty with kinematic alignment using specific instruments versus mechanical alignment in varus knees

**DOI:** 10.3389/fsurg.2022.1097302

**Published:** 2023-01-18

**Authors:** Liang Wen, Zhiwei Wang, Desi Ma, Xiaoxiong Zhao

**Affiliations:** Department of Orthopedics, Beijing Chaoyang Hospital, Capital Medical University, Beijing, China

**Keywords:** kinematic alignment, mechanical alignment, osteoarthritis, knee, arthroplasty, follow-up studies

## Abstract

**Background:**

The kinematic alignment technique, as one of the alignment options for total knee arthroplasty, has attracted increasing attention from orthopedic surgeons and has been increasingly performed in the most populous countries in the world. The purpose of this study is to explore and compare the early clinical outcomes of total knee arthroplasty with KA using specific instruments vs. mechanical alignment in our nation.

**Methods:**

A retrospective analysis was performed on patients who underwent unilateral total knee arthroplasty for knee osteoarthritis with varus deformity. Depending on the alignment method, patients were divided into a kinematically aligned total knee arthroplasty (KA-TKA) group and a mechanically aligned total knee arthroplasty (MA-TKA) group. The hip-knee-ankle (HKA) angle before and after surgery, the knee joint clinical score (KS-C), the knee joint functional score (KS-F) and the forgotten joint score (FJS) at 3 months and 2 years after surgery were recorded and statistically analyzed.

**Results:**

A total of 126 patients were enrolled, including 65 in the KA-TKA group and 61 in the MA-TKA group. The mean follow-up period was 30.8 months. The postoperative HKA angle was not significantly different at the 2-year follow-up between the two groups (*P* > 0.05). The KS-C, KS-F and FJS scores in the KA-TKA group were higher than those in the MA-TKA group at 3 months after surgery, and the difference was statistically significant (*P* < 0.05). At the 2-year follow-up, the KS-C, KS-F and FJS scores in the KA-TKA group were higher than those in the MA-TKA group, and the difference in the KS-C and FJS scores was statistically significant (*P* < 0.05).

**Conclusion:**

Patients who underwent KA-TKA had a postoperative lower limb alignment similar to that of those who underwent MA-TKA. The clinical outcomes of KA-TKA were superior to those of MA-TKA in terms of clinical performance, knee function and subjective sensation up to 2 years after surgery.

## 1. Introduction

Total knee arthroplasty (TKA) is one of the most effective surgical procedures for the treatment of end-stage osteoarthritis of the knee, and the number of patients undergoing TKA is increasing significantly each year worldwide (1). At present, knee prostheses in patients undergoing TKA are usually implanted using the mechanical alignment (MA) technique, by which the femoral and tibial components are perpendicular to the mechanical axis of the lower limb to achieve a neutral hip-knee-ankle (HKA) angle, thereby optimizing load distribution in the medial and lateral compartments and reducing the risk of wear and loosening (2). Although the MA philosophy can improve the prosthesis survival rate and patient satisfaction (3, 4), approximately 20% of patients are not satisfied with their prosthetic knees, and the widely accepted reasons for patient dissatisfaction are mainly postoperative pain and poor knee function (5, 6). Although computer-assisted or robotics-assisted surgery has improved the accuracy of mechanical alignment in recent years, the problems mentioned above have not been solved (7).

In recent years, kinematically aligned total knee arthroplasty (KA-TKA) has attracted increasing attention from knee surgeons due to its excellent clinical outcomes (8, 9). Unlike traditional MA philosophy, which pursues neutral alignment, KA-TKA is an individualized alignment method with the aim of restoring patients' prearthritic joint line and lower extremity alignment (10). KA reestablishes the natural knee flexion and extension axis by restoring the joint line inclination, and the distal and posterior femoral condyle and proximal tibia are resurfaced equally both in thickness and orientation by prostheses (11). Seldom ligament release is required to restore the patient's prearthritic soft tissue tension and to contribute to a more natural feeling of the knee joint.

To achieve a more accurate and convenient KA-TKA, a set of KA-specific instruments was developed from traditional TKA manual instruments by the authors' team and applied for implantation of the corresponding commercial knee prosthesis in this study. The KA-TKA surgical techniques using the KA-specific instrument were described, and the early clinical results of KA-TKA and MA-TKA were compared in this study.

## 2. Materials and methods

### 2.1. Patients

Patients who underwent unilateral total knee arthroplasty for knee osteoarthritis with varus deformity between October 2018 and September 2020 at Beijing Chaoyang Hospital, Capital Medical University, were retrospectively analyzed. Patients diagnosed with grade III or IV osteoarthritis based on the Kellgren-Lawrence classification, operated on under general anesthesia, and who were followed up for more than 24 months after surgery were included. Patients with inflammatory arthrosis, traumatic osteoarthritis, valgus knee, flexion contracture greater than 20° without posterior osteophytes, patellofemoral disorders, previous collateral ligaments or posterior cruciate ligament injury, revision surgery, psychiatric disorders and medication were excluded in the present study. The study was approved by the ethics committee of our hospital, and all patients signed consent forms.

Based on the above inclusion and exclusion criteria, a total of 130 patients were included in this study, with a follow-up period of 24–36 months. Sixty-five consecutive patients in the KA-TKA group underwent surgery by a senior surgeon (LW) from October 2019 to September 2020 using a set of KA-specific instruments developed by the authors' research team. Sixty-five patients who underwent MA-TKA from October 2018 to September 2019, which was also performed by the same senior surgeon (LW), were randomly selected into the MA-TKA group. All prostheses were fixed with cement, and a posterior cruciate retention (CR) prosthesis (Gemini MK II, LINK, Germany) was used for all patients.

### 2.2. Surgical techniques

#### 2.2.1. MA-TKA

**Figure 1:**
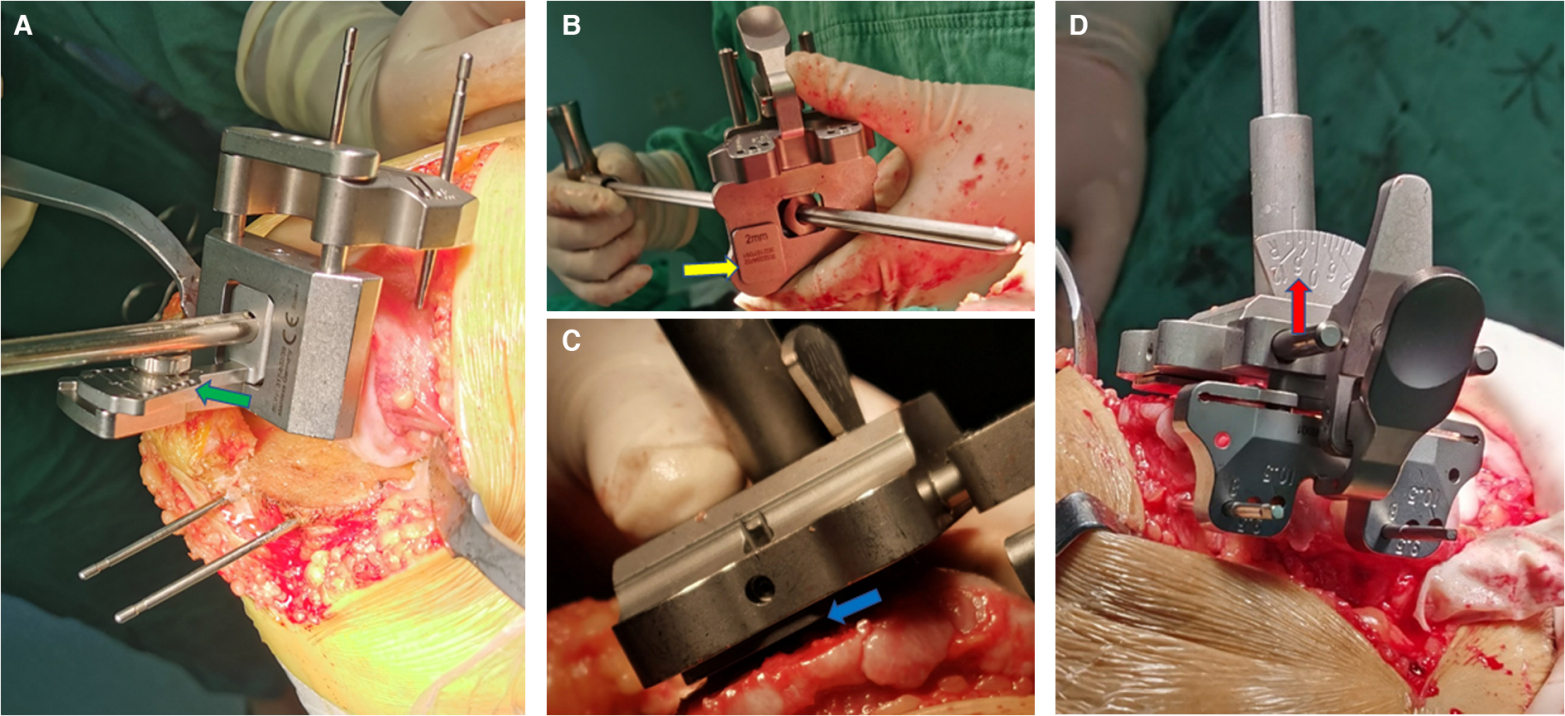
Placement and fixation of the intramedullary positioning guide plate for distal femoral bone resection in coronal plane (**A** for MA; **B–D** for KA). (**A**) The standard MA femoral intramedullary guided assembly, the valgus cutting angle was set at 6° with a chuck (green arrow). (**B**) An insert (yellow arrow) with 2 mm thickness prefabricated at the medial side on the distal femoral guide plate of the specific KA instrument. (**C**) The distal femoral guide plate with the insert appressed against the worn distal articular surface of the medial condyle for compensation of cartilage wear (blue arrow); (**D**) The cutting block of the specific KA instrument can be rotated freely up to ±12° on the coronal plane (red arrow), which is enough for varus-valgus adjustment according to native distal femur joint line.

**Figure 2:**
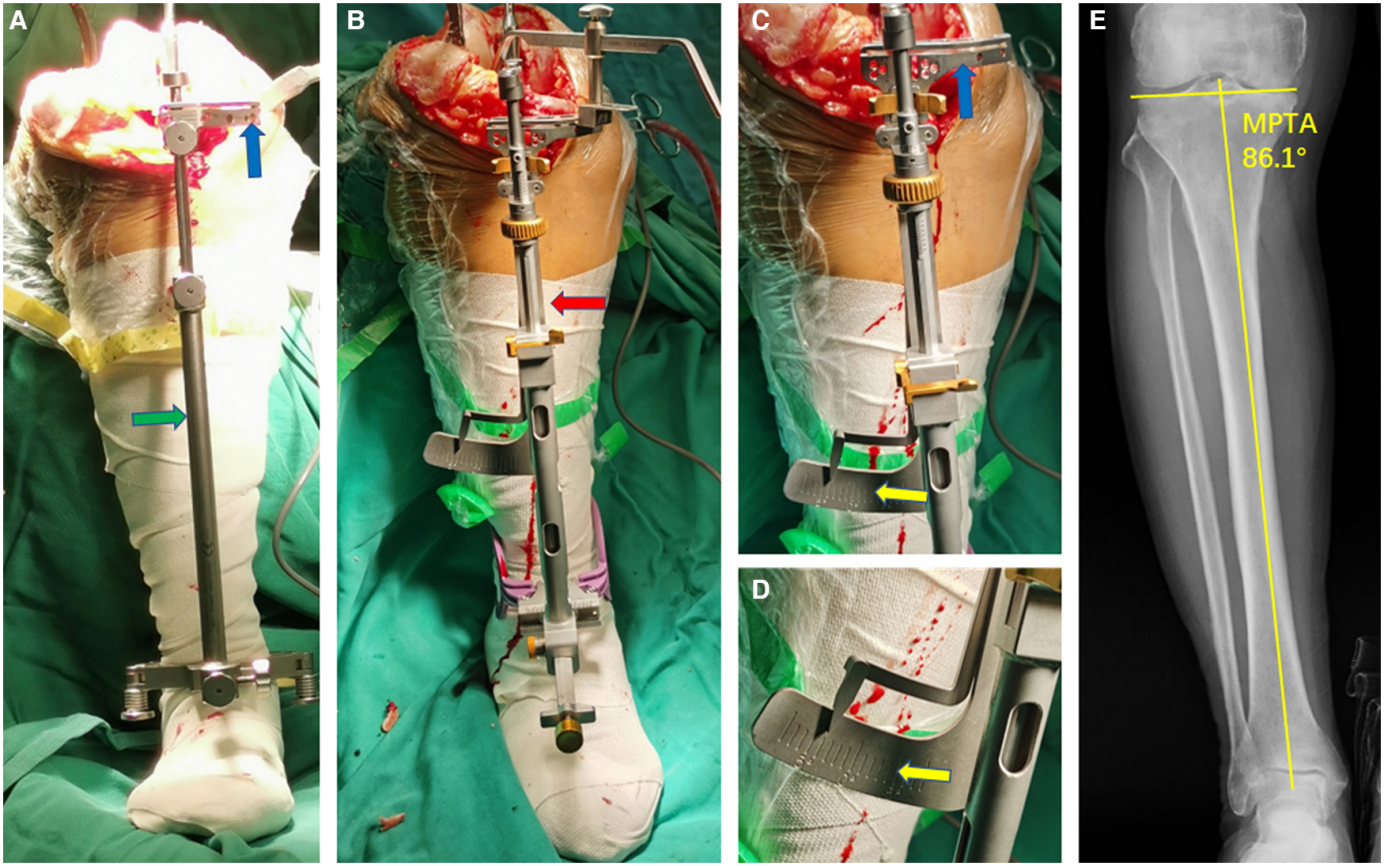
Tibial extramedullary assembly and its application (**A** for MA; **B–D** for KA). (**A**) Standard MA tibial extramedullary assembly. The extramedullary reference rod (green arrow) was set parallel to the mechanical axis of the tibia, and the proximal tibia cutting block (blue arrow) was perpendicular to the tibial mechanical axis. (**B**) The extramedullary reference rod of the specific KA instrument (red arrow) was also set parallel to the mechanical axis of the tibia. (**C**) The extramedullary assembly of the specific KA instrument has an angular scale (yellow arrow) in conjunction with the cutting block (blue arrow), which guaranteed an equal-angle linkage mechanism. (**D,E**) The angular scale (yellow arrow) can be adjusted in the range of ±8° in varus or valgus. Usually, the angular scale was set in accordance with preoperative MPTA to restore the native joint line inclination in the coronal plane.

**Figure 3:**
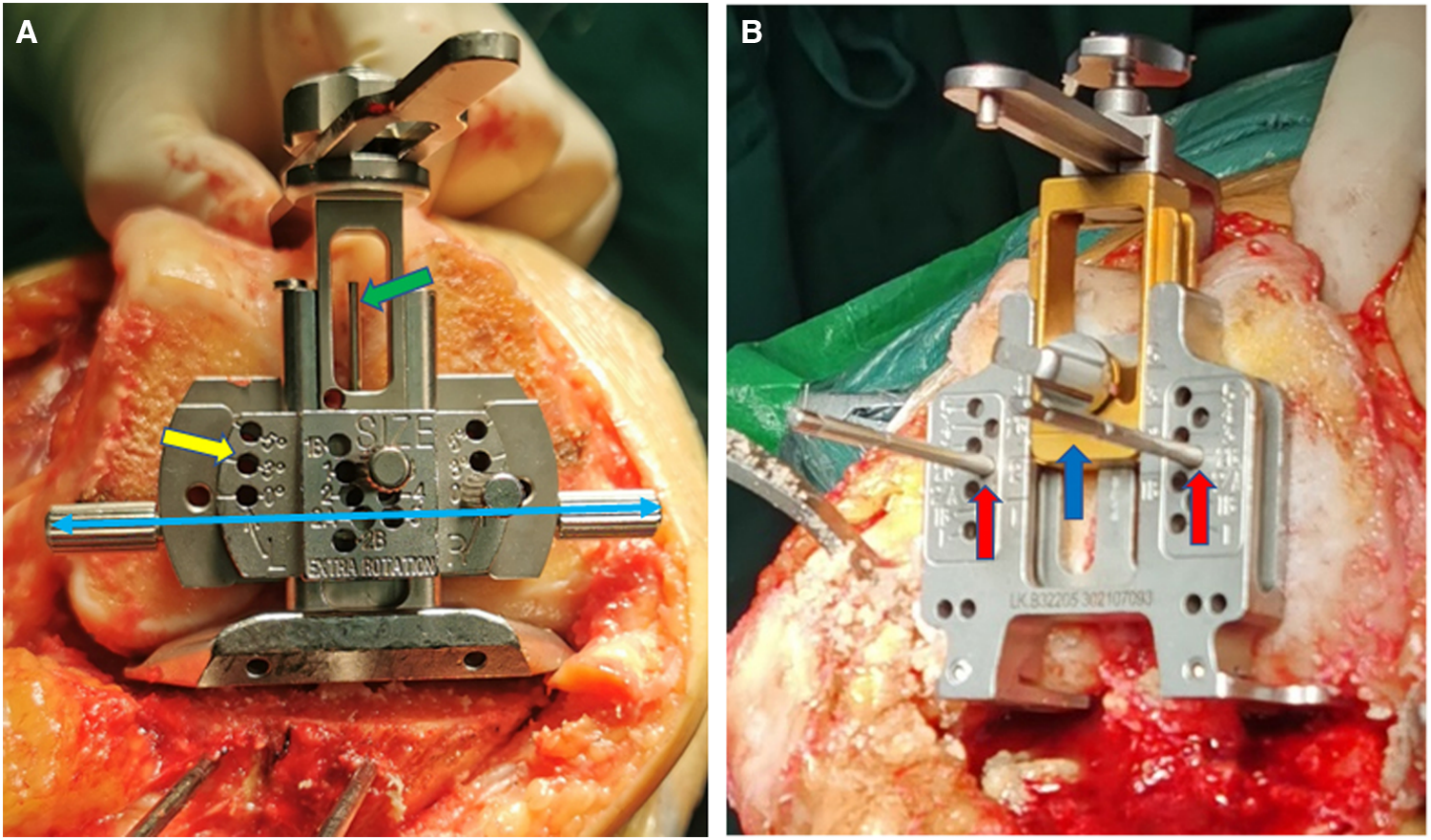
Femoral sizing and rotational positioning (**A** for MA; **B** for KA). (**A**) Standard MA rotational positioning device. The measured resection technique was applied for the external rotational positioning in the distal femur depending on Whiteside line (green arrow), transcondylar line (blue double arrow), or setting to 3° external rotation (yellow arrow). (**B**) Femoral sizer and positioning device of the specific KA instrument. Place the femoral sizer against the resected surface of the distal femur with the posterior feet of the sizer contacting the posterior condyles. The gold slider (blue arrow) is used to size the femur, and the pairs of pin holes (red arrow) on the silver component are posterior reference design parallel to the PCA to ensure an equal resection of the posterior condyles.

A pneumatic tourniquet was used throughout the procedure. A medial parapatellar approach was used to expose the articular cavity with partial preservation of the infrapatellar fat pad. The anterior cruciate ligament was excised if it was *in situ*, while the posterior cruciate ligament was preserved. The distal femoral cut was guided intramedullary and performed at 6° of valgus (Figure 1A). The proximal tibia cut was positioned extramedullary and perpendicular to the tibial mechanical axis with a 7° posterior slope (Figure 2A). The measured resection technique was applied for external rotational resection in the distal femur (Figure 3A). Soft tissue release was performed to balance the flexion and extension gaps if necessary.

#### 2.2.2. KA-TKA

The exposure was the same as that for MA-TKA. Specific KA instruments were applied. The distal femoral cut was also guided intramedullary but did not set a certain valgus angle. Metal inserts (2 mm) prefabricated on the distal femoral guide plate were used for compensation of 2 mm of cartilage wear of the distal femur (12) (Figures 1B–D), usually at the medial side in varus knees. After resecting the distal femur, a femoral sizer was placed parallel to the posterior condyle's axis (PCA) (Figure 3B) and then finished posterior, anterior, and chamfer bone resection with 4 in 1 cutting block (Figure 3). After all bone resections were completed on the femoral side, each bone cut piece was measured with a caliper to check whether their thicknesses were the same as the corresponding parts of the femoral component after compensating for cartilage wear and the kerf of the saw blade. If there was a deviation of any pieces more than 0.5 mm, a manual additional recut was performed at the corresponding condyle for under resection or filling the gap of over resection with bone cement during cementation, with the aim of reducing the deviation to less than 0.5 mm. A specific extramedullary tibial assembly was applied to the resection at the proximal tibia. The extramedullary reference rod was placed parallel to the mechanical axis of the tibia. An angular scale in conjunction with the cutting block attached to the assembly (Figure 2B) was used to set the varus-valgus cut so that it aligned with the native joint line orientation, that is, to restore the native medial proximal tibial angle (MPTA) (Figures 2C–E). The inclined cut in the sagittal plane was set in line with the native posterior tibial slope of the medial plateau, and the axial rotation was set parallel to the long axis of the ellipse of the lateral tibial plateau (13). After completing all resections, if there was a medial-lateral imbalance in the extension gap, manual varus-valgus recut of the tibial plateau was executed to balance the extension gap. Soft tissue release is always not performed.

### 2.3. Perioperative management and postoperative follow-up

The intraoperative cocktail analgesia regimen and articular administration of tranexamic acid were completely identical between the two groups. Prophylactic antibiotics were applied within 24 h after surgery. Rivaroxaban was administered to prevent deep vein thrombosis until 14 days after surgery. Ankle pump exercises were started immediately after surgery, and active and passive knee flexion and extension activity and quadriceps muscle strength exercises were started on the first day after surgery. All patients underwent rehabilitation independently at home according to the unified protocol after discharge. Outpatient follow-up was performed at 3 months and 2 years after surgery.

### 2.4. Assessment parameters

The patients' body mass index (BMI) was calculated. HKA before and after surgery was measured. The preoperative knee joint clinical score (KS-C) and knee joint functional score (KS-F) and the postoperative KS-C, KS-F and forgotten joint score (FJS) at 3 months and 2 years were recorded. Perioperative complications were also recorded.

### 2.5. Statistical analysis

SPSS (SPSS 19.0, IBM Inc., USA) was applied for statistical analysis. Continuous variables conforming to the normal distribution are presented as the mean ± standard deviation. The preoperative data of both the KA-TKA and MA-TKA groups and the postoperative HKA were compared with Student's *t* test. Sex distribution and Kellgren-Lawrence classification of the two groups were analyzed by the chi-square test. The KSS clinical scores, KSS functional scores and FJS scores between the KA-TKA and MA-TKA groups before surgery and 3 months and 2 years after surgery were analyzed with repeated-measures ANOVA. Greenhouse‒Geisser's adjustment results prevailed if Mauchly's test of sphericity was not reached. Pairwise comparisons of *post hoc* tests were performed using the Bonferroni test. The level of significance was set at *P* < 0.05.

## 3. Results

### 3.1. Patient demographics and preoperative assessment parameters

All 65 patients in the KA-TKA group and 61 patients in the MA-TKA group had full follow-up data, while 4 patients in the MA-TKA group were excluded for absence at the 2-year follow-up. The mean follow-up period was 30.8 months (24–36 months). There were no significant differences between the KA-TKA and MA-TKA groups in terms of sex, Kellgren-Lawrence classification, age, BMI, HKA, KSS-C or KSS-F before surgery (Table 1).

**Table 1:** Comparison of preoperative data of patients in the KA-TKA and MA-TKA groups (mean ± SD).

	Sex M/F	K-L III/IV	Age	BMI	HKA	KSS-C	KSS-F
KA	19/46	48/17	70.6 ± 6.4	27.3 ± 3.4	172.4 ± 4.0	49.1 ± 15.7	30.5 ± 17.6
MA	21/40	49/12	71.2 ± 7.1	27.2 ± 3.7	172.8 ± 4.1	47.6 ± 13.8	27.1 ± 14.3
	*χ*^2^ = 0.392	*χ*^2^ = 0.746	*t* = 0.51	*t* = 0.02	*t* = 0.53	*t* = 0.63	*t* = 1.21
*P*	0.531	0.388	0.611	0.987	0.599	0.53	0.228

### 3.2. Comparison of postoperative assessment parameters between the KA-TKA and MA-TKA groups

**Figure 4:**
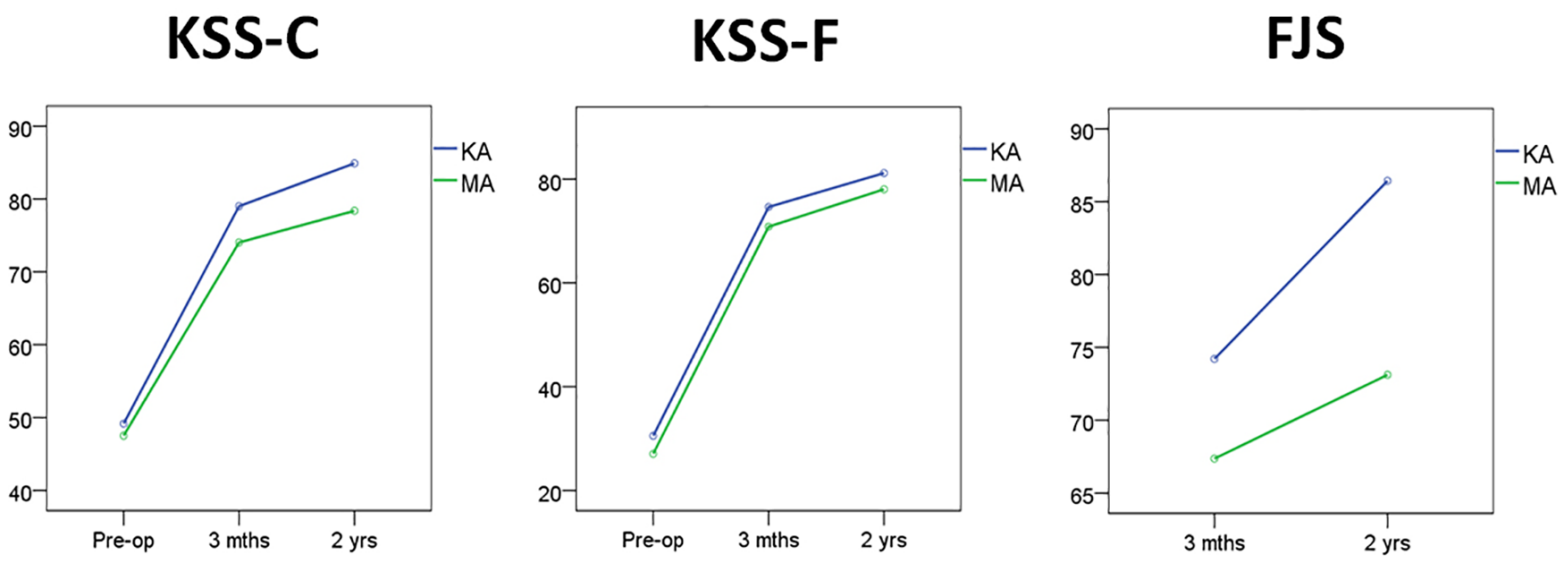
The trends of KSS-C, KSS-F and FJS before and after surgery. KA, kinematic alignment; MA, mechanical alignment; KSS-C, knee joint clinical score; KSS-F, knee joint functional score; FJS, forgotten joint score.

At the end of the 2-year follow-up after surgery, the KHA was 177.3 ± 1.9° in the KA group and 177.8 ± 1.4° in the MA group, without a significant difference between the two groups (*t* = 1.36, *P* = 0.176). After preliminary data analysis, KSS-C, KSS-F and FJS did not conform to Mauchly's test of sphericity. Therefore, Greenhouse‒Geisser's adjustment results prevailed. Repeated measures ANOVA results showed that the interactions of KSS-C and FJS in groups and time were significant, while KSS-F was not (Table 2). The simple effect test of groups showed that the postoperative KSS-C, KSS-F and FJS scores at the 3-month follow-up were all significantly different, and the results of the KA group were better than those of the MA group. At the end of the 2-year follow-up, there were significant differences in the KSS-C and FJS between the two groups, with the results of the KA group being better than those of the MA group, while there was no significant difference between the two groups in terms of the KSS-F (Table 3, Figure 4). The simple effect test of Time showed that there were significant differences in KSS-C, KSS-F and FJS scores in both the KA and MA groups at different time points (Table 4). Further pairwise comparisons showed that KSS-C, KSS-F and FJS increased chronologically in both the KA and MA groups before surgery and 3 months and 2 years after surgery, and the differences reached the level of significance (*P* = 0.000).

**Table 2:** Comparison of postoperative data between patients in the KA-TKA and MA-TKA groups (mean ± SD).

	KSS-C			KSS-F			FJS	
	Before surgery	At 3-month follow-up	At 2-year follow-up	Before surgery	At 3-month follow-up	At 2-year follow-up	At 3-month follow-up	At 2-year follow-up
KA group	49.16 ± 15.71	79.01 ± 6.44	84.89 ± 8.59	30.54 ± 17.60	74.61 ± 9.50	81.15 ± 9.51	74.20 ± 7.38	86.43 ± 9.65
MA group	47.51 ± 13.78	74.03 ± 6.18	78.37 ± 9.32	27.06 ± 14.27	70.82 ± 8.96	78.03 ± 10.62	67.36 ± 9.82	73.12 ± 13.82
Interaction in groups	*F* = 6.34, *P* = 0.013, Partial *η*^2^ = 0.049			*F* = 3.13, *P* = 0.079, Partial *η*^2^ = 0.025			*F* = 32.05, *P* = 0.000, Partial *η*^2^ = 0.205	
Interaction in time	*F* = 1505.35, *P* = 0.000, Partial *η*^2^ = 0.924^a^			*F* = 2302.80, *P* = 0.000, Partial *η*^2^ = 0.949^a^			*F* = 320.75, *P* = 0.000, Partial *η*^2^ = 0.721^a^	
Interaction in groups & time	*F* = 7.20, *P* = 0.003, Partial *η*^2^ = 0.055^a^			*F* = 0.086, *P* = 0.853, Partial *η*^2^ = 0.001^a^			*F* = 41.60, *P* = 0.000, Partial *η*^2^ = 0.251^a^	

^a^ Greenhouse-Geisser's adjustment results prevailed.

**Table 3:** The statistical results of the simple effect test of groups between KA and MA.

	KSS-C	KSS-F	FJS
Before surgery	*F* = 0.396, *P* = 0.530, Partial *η*^2^ = 0.003	*F* = 1.470, *P* = 0.228, Partial *η*^2^ = 0.012	N.A.
At 3-month's follow-up	*F* = 19.583, *P* = 0.000, Partial *η*^2^ = 0.136	*F* = 5.316, *P* = 0.023, Partial *η*^2^ = 0.41	*F* = 19.687, *P* = 0.000, Partial *η*^2^ = 0.137
At 2-year's follow-up	*F* = 16.669, *P* = 0.000, Partial *η*^2^ = 0.119	*F* = 3.028, *P* = 0.084, Partial *η*^2^ = 0.024	*F* = 39.702, *P* = 0.000, Partial *η*^2^ = 0.243

**Table 4:** The statistical results of the simple effect test of time in the KA and MA groups.

	KSS-C	KSS-F	FJS
KA group	*F* = 959.854, *P* = 0.000, Partial *η*^2^ = 0.915	*F* = 955.883, *P* = 0.000, Partial *η*^2^ = 0.940	*F* = 306.418, *P* = 0.000, Partial *η*^2^ = 0.712
MA group	*F* = 455.759, *P* = 0.000, Partial *η*^2^ = 0.881	*F* = 883.877, *P* = 0.000, Partial *η*^2^ = 0.935	*F* = 63.638, *P* = 0.000, Partial *η*^2^ = 0.339

### 3.3. Postoperative complications

No major complications, such as symptomatic deep vein thrombosis, pulmonary embolism or periprosthetic infection, occurred during the follow-up period. There were two cases of delayed wound healing caused by fat liquefaction and exudation in each group.

## 4. Discussion

To the best of our knowledge, this is the first study in mainland China to report comparative results between KA-TKA and MA-TKA with a postoperative follow-up of 2 years. Our study found that the patients who underwent KA-TKA had higher knee function scores and higher FJS scores than patients with MA-TKA at 3 months and 2 years after surgery. The results showed that the patients with KA-TKA had more natural activity of their prosthetic joint subjectively and a higher level of satisfaction. In addition, the postoperative alignment of the lower limbs in the KA-TKA group was similar to that in the MA-TKA group.

In this study, we found that KA-TKA was associated with better pain relief, knee function and satisfaction at the 3-month follow-up than MA-TKA, and these outcomes lasted for at least 2 years after surgery, which may be due to KA's respect for individual anatomy and the restoration of the native profile of the prearthritic knee joint. Unlike MA, the goal of KA is to match the orientation of the prosthetic component to each patient's own anatomy (14). By adjusting the direction and amount of resection of the distal femur, posterior condyle and tibial plateau, the goal of KA is to restore the orientation of the native joint line, the alignment of the lower extremity and the laxity of the joint after TKAs to the status before the onset of osteoarthritis, thus maximizing the possibility of reproducing native knee kinematics (15). The bone resection in KA-TKA is not guided by anatomical landmarks such as the coronal axis of the lower extremity or the transcondylar axis but rather by replacing the resected portion equivalently with the prosthetic components and reproducing a native-like prearthritis knee surface (16), which restores the natural anatomy of the knee joint and preserves the soft tissue envelope, thus minimizing ligament and soft tissue release and interference (17).

These findings were consistent to some extent with some previous studies. Matsumoto et al.'s RCTs showed greater improvement in flexion angle, knee function scores and patient satisfaction after KA-TKA than after MA-TKA (18). The RCTs by MacDessi et al. demonstrated that the application of the KA alignment technique in TKA resulted in a more significant improvement in the quantitative balance of the knee (19). A comparative study of bilateral TKA in the same patient by McEwen et al. found similar clinical outcomes on the KA-TKA side vs. the MA-TKA side 2 years after surgery, but patients subjectively preferred the KA knee (20). Relevant meta-analyses have also been summarized comprehensively in recent years. A meta-analysis by Liu et al. (21) showed that KA-TKA was superior to MA-TKA in terms of KSS scores, WOMAC scores, knee flexion, and functional outcomes, which was consistent with the results of several previously published meta-analyses (22–24). What is different from the above study was that Young's (2) and Yeo's (25) study showed no difference in clinical outcomes, imaging performance, or gait analysis between KA-TKA and MA-TKA at the 2-year postoperative follow-up. It is precisely because of these inconsistent results that more doctors and patients from different regions in the world are needed to participate in the research field of TKA alignment strategy.

The present study showed that there was no significant difference in the coronal alignment of the lower extremity between the KA-TKA and MA-TKA groups after surgery, which is consistent with the results of some previous studies (26, 27). The main difference between the KA and MA philosophy is whether the patient's premorbid joint line inclination is restored. The result of no difference in the coronal alignment of the lower extremity between the two groups after surgery can be explained by the more valgus femoral component and the more varus tibial component placed in KA-TKA than in MA-TKA (28). The main controversial point of KA technology is the concern about the potential increase in risk of loosening of the prosthesis due to the nonvertical alignment of the prosthesis components. Some clinical studies in recent years have shown that an alignment deviation of less than 0 ± 3° 20 years after MA-TKA did not guarantee a lower rate of prosthesis failure (29), with no difference in the migration of the tibial prosthesis components in KA-TKA and MA-TKA between 2 and 9 years postoperatively, and this kind of displacement was independent from alignment (30). Although the prosthesis survival rate at 10 years after KA-TKA was not inferior to that after MA-TKA (31), further long-term follow-up studies and basic research evidence on KA-TKA are still needed to dispel this concern.

There were some limitations in this study. The study was a single-center, nonrandomized controlled study with a possible bias in patient selection. Patients with valgus knees were not included in this study, so the results of this study have little reference value for patients with valgus knee deformities. The KA-TKA in this study was performed with specific instruments developed by the authors' team, and a CR prosthesis was used in all cases in both groups, which was not applicable for other surgical instruments or other types of prostheses.

## 5. Conclusion

The clinical outcomes of KA-TKA were superior to those of MA-TKA in terms of clinical performance, knee function and subjective sensation up to 2 years after surgery. Interestingly, the alignment of the lower extremity remained somewhat varus after surgery in varus knees, regardless of the alignment strategy.

## Data Availability

The original contributions presented in the study are included in the article/Supplementary Material, further inquiries can be directed to the corresponding author.
